# Relationship between happiness and tobacco smoking among high school students

**DOI:** 10.4178/epih.e2018009

**Published:** 2018-03-24

**Authors:** Maryam Ataeiasl, Parvin Sarbakhsh, Hossein Dadashzadeh, Christoph Augner, Masoumeh Anbarlouei, Asghar Mohammadpoorasl

**Affiliations:** 1 Department of Statistics and Epidemiology, Faculty of Health, Tabriz University of Medical Sciences, Tabriz, Iran; 2 Research Center of Psychiatry and Behavioral Sciences, Tabriz University of Medical Sciences, Tabriz, Iran; 3 Institute for Human Resources Research in Health Care, University Clinics of the Paracelsus Medical University, Salzburg, Austria; 4 Tabriz Health Services Management Research Center, Tabriz University of Medical Sciences, Tabriz, Iran

**Keywords:** Cigarette smoking, Hookah smoking, Adolescents, Happiness, Iran

## Abstract

**OBJECTIVES:**

Recent research has described negative relationship between happiness and habitual smoking among adolescents. No study of this relationship has been conducted among Iranian adolescents. The aim of the present study was to characterize the relationship between happiness and cigarette or hookah smoking among a sample of high school students.

**METHODS:**

A sample of 1,161 10th-grade students in Tabriz (northwest Iran) was selected by multi-stage proportional cluster sampling. Participants completed a self-administered multiple-choice questionnaire including information on cigarette smoking, hookah smoking, happiness score, substance abuse, self-injury, general risk-taking behavior, attitudes towards smoking, socioeconomic information, and demographic characteristics. An ordinal logistic regression model was used for data analysis.

**RESULTS:**

It was found that 5.9 and 5.0% of students were regular cigarette smokers and regular hookah smokers, respectively. After controlling for potential confounders, higher happiness scores were found to protect students against more advanced stages of cigarette smoking (odds ratio [OR], 0.98; 95% confidence interval [CI], 0.97 to 0.99; p=0.013). However, no significant relationship was found between happiness scores and hookah smoking status (OR, 1.01; 95% CI, 0.97 to 1.02; p=0.523).

**CONCLUSIONS:**

Happiness scores were associated with less advanced stages of habitual cigarette smoking among high school students. Our findings underscore the necessity of conducting longitudinal or interventional studies aiming to determine the effects of enhancing happiness on preventing the transition through the stages of cigarette and hookah smoking.

## INTRODUCTION

The negative effects of smoking on health are well established. It is estimated that approximately 50.0% of smokers will die due to their behavioral habit [1]. The second greatest risk factor of the global burden of disease is tobacco use, which was estimated to be the cause of 6.1 million deaths and 143.5 million cases of disability in 2013 [2]. Tobacco use starts during adolescence and youth [1,3]. Smoking is a learned behavior beginning in adolescence [1], and individuals who do not begin smoking during adolescence are less likely to smoke in adulthood [1]. Decreasing the smoking rate among adolescents is a major public health priority [4]. In Iran, the prevalence of smoking among adolescents has been reported to range from 2.5 to 17.0% [5-8]. Studies revealed an increasing prevalence of smoking among Iranian adolescents [9]. Moreover, it seems that the speed of becoming a smoker in Iranian adolescents is high [5,10].

Happiness is a fuzzy concept. Associated concepts include well-being, quality of life, flourishing, and contentment. Confirmation of the effects of positive psychological variables such as happiness on well-being has led to an increased focus on positive psychology in recent years. Recent research indicates that increased happiness has numerous health advantages, such as a reduced risk of mortality, morbidity, and disability. Happiness is important among adolescents due to its effect on their success in the future [11,12].

Recent studies have indicated that happiness and smoking are negatively related in adolescents [13]. Adolescents who experience high levels of happiness typically exhibit fewer emotional and behavioral problems [14]. Heizomi et al. [15] showed that adolescents with a history of smoking over the past 6 months had significantly lower well-being scores.

Studies conducted on smoking among adolescents have focused on investigating and determining the prevalence and risk factors of smoking. Due to the importance of paying attention to positive psychology, especially happiness, the present study aimed to characterize the relationship between happiness and cigarette or hookah smoking among a sample of high school students.

## MATERIALS AND METHODS

This cross-sectional school-based study was the first phase of an interventional longitudinal study of smoking prevention through happiness enhancement. We studied a sample of 10th-grade students in Tabriz (northwest Iran) during February 2017. The reason for limiting the subjects to 10th-grade students was the potential to track individuals in the next phases of the longitudinal study. The sampling method was multi-stage proportional cluster sampling. First, 11 high schools were selected randomly among all high schools in the city of Tabriz. Then, 43 classes were selected as clusters based on the gender (since women and men are taught in separate classrooms in the education system in Iran) and major of students. A total of 1,161 students participated in the study.

After we clarified the research objectives, all students provided oral informed consent and were included in the study. Participants completed a self-administered multiple-choice anonymous questionnaire in the classroom. The questionnaire was approved by the Ethics Committee of Tabriz University of Medical Sciences (ethical code: IR.TBZMED.REC.1396.188) and the Research Committee of the East Azerbaijan Province Education Organization.

The aim of the questionnaire was to obtain information on cigarette smoking, hookah smoking, happiness score, history of substance abuse, experience of self-injury, general risk-taking behavior, attitudes towards smoking, socioeconomic information, and demographic characteristics.

Happiness was measured by the Oxford Happiness Questionnaire, which contains 29 statements that respondents evaluate on a scale from 1 (strongly disagree) to 6 (strongly agree). A few examples of these items are “Life is good,” and “I am well satisfied about everything in my life.” The happiness score ranges from 29 to 174, where higher scores indicate a higher level of happiness. The reliability and validity of the Persian version of this questionnaire among students in Iran have been confirmed [16].

Cigarette smoking status was assessed by a validated algorithm for adolescent smoking [17]. Following classifications used in previous studies [5,10], cigarette smoking status was subdivided into the following 3 stages:

(1) Never cigarette smoker: Students who have never tried cigarettes.

(2) Cigarette experimenter: Students who indicated having tried cigarettes or having smoked fewer than 100 cigarettes.

(3) Regular cigarette smoker: Students who indicated having smoked 100 cigarettes or more in their lifetime.

Hookah smoking was measured using a multiple-choice question with the answers of never having smoked hookah, only having tried hookah, occasionally smoking hookah, smoking hookah at least once a month, and smoking hookah at least once a week. The students were then classified into the following 3 statuses of hookah smoking:

(1) Never hookah smoker: Students who have never smoked hookah (even a puff).

(2) Hookah experimenter: Students who have tried hookah smoking or have smoked occasionally.

(3) Regular hookah smoker: Students who smoked hookah at least once a month.

General risk-taking behavior was assessed by the following question: “Do you enjoy doing things that are a dangerous or risky?” Respondents who answered “Yes” were classified as engaging in risk-taking behavior.

Any use of substances such as cannabis, opium, ecstasy, or methamphetamines in their lifetime was considered a “Yes” response for the substance abuse variable.

Attitudes toward cigarette smoking were assessed by a validated and reliable 6-item questionnaire that has been used in previous studies in Iran [5,10]. The potential range of scores on this questionnaire was −12 to 12, with higher scores indicating positive attitudes.

Using principal component analysis, socioeconomic status was measured in terms of the father’s education, the mother’s education, family assets, and family income. This measure graded students into very high, high, middle, lower, and very low levels of socioeconomic status.

Because of the cluster sampling method, survey analysis was used for all variables. The chi-square test and one-way analysis of variance were used for univariate data analysis. Because the dependent variables had 3 levels (cigarette smoking stages and hookah smoking status), an ordinal logistic regression model was used for the multivariate analysis. All analyses were done using Stata version 14 (StataCorp., College Station, TX, USA).

## RESULTS

The participation rate was 97.6% (1,133 of 1,161). The mean age of students was 15.48± 0.50 years (range, 14-17 years). Of the students, 567 (50.0%) were men and 566 (50.0%) were women. The results of the study indicated that 890 of the students (78.7%) were never smokers, 174 (15.4%) were experimenters, and 67 (5.9%) were regular smokers. In term of hookah smoking, 664 (61.5), 361 (33.5), and 54 (5.0%) students were never, experimenter, and regular hookah smokers, respectively.

Table 1 shows the frequency distribution of cigarette smoking by demographic features and other variables. Gender, general risktaking behaviors, experience of self-injury, substance abuse, having smoker friend(s), having smoker(s) in the family, hookah smoking status, living with parents, attitudes toward smoking, average grades in the previous year, and the happiness score were significantly related to cigarette smoking.

Table 2 shows the frequency distribution of hookah smoking by demographic features and other variables. Gender, general risktaking behaviors, experience of self-injury, substance abuse, having smoker friend(s), having smoker(s) in the family, higher socioeconomic status, cigarette smoking status, attitude toward smoking, average grades in the previous year, and the happiness score were significantly associated with hookah smoking.

We evaluated the relationships of happiness with the stage of cigarette smoking status and with hookah smoking status using 2 ordinal logistic models that adjusted for potential confounders (Table 3). After controlling for general risk-taking behaviors, experience of self-injury, substance abuse, smoker friend(s), smoker(s) in the family, hookah smoking status, living with parents, attitudes toward smoking, and average grades in the previous year, higher happiness scores protected students from more advanced stages of cigarette smoking (odds ratio [OR], 0.98; 95% confidence interval [CI], 0.97 to 0.99; p=0.013). This means that for each 1-unit increase in the happiness score, the odds of being at a higher cigarette smoking stage versus a lower cigarette smoking stage was 0.98 times lower, controlling for the other variables in the model. After controlling for gender, general risk-taking behaviors, experience of self-injury, substance abuse, smoker friend(s), smoker(s) in the family, socioeconomic status, cigarette smoking status, attitude toward smoking, and average grades in the previous year, no significant association was found between the happiness score and hookah smoking status (OR, 1.01; 95% CI, 0.97 to 1.02; p=0.523).

## DISCUSSION

The present study investigated the relationships of happiness with stages of cigarette smoking and hookah smoking status among high school students. Univariate analysis indicated a significant negative relationship between the happiness score and smoking stage; that is, the more advanced the stage of smoking, the lower the happiness score was found among the students. Furthermore, the ordinal logistic regression model showed that when controlling for confounders, a single-unit increase in the average happiness score, was associated with less chance of being at a higher stage of cigarette smoking. The negative relationship observed between cigarette smoking and happiness in this study is consistent with previous research [13,18]. As a possible explanation for this relationship, it could be hypothesized that a reduced experience of negative feelings in adolescents would be associated with a reduced inclination toward experiencing or continuing smoking, as smoking can be seen as a mood modulator. A study in South Korea demonstrated a negative relationship between happiness and smoking in both genders, which was explained by the proposal that adolescents who were discontent with their life smoked more than those who had not experienced this feeling [13]. Furthermore, this relationship was stronger among girls, which supports the hypothesis that girls are more sensitive to problems and use smoking as a mood changer [13,19]. Cable et al. [20] found that higher happiness scores among adolescents were longitudinally associated with resistance against smoking. Several longitudinal studies have suggested that serious repetitive and negative feelings are prospective predictors of increased smoking, and heavy smoking can predict the subsequent progress of depression symptoms. According to the findings of a study in 9 countries of the former Soviet Union, in all the countries except Kyrgyzstan, individuals with smoking experience and non-smokers were significantly happier than routine smokers [21]. However, some studies have reported a positive relationship between smoking and happiness [15,22]. Moreover, a study showed that smoking and happiness were associated among people in France; however, in Japan, France, and England, individuals with a low rate of smoking showed higher happiness scores [23]. In the present study, the univariate analysis found a significant relationship between happiness and hookah smoking, in which never hookah smokers had higher happiness scores than students who had experienced hookah or smoked hookah weekly or monthly. However, in the multivariate analysis, when controlling for other confounding variables in the ordinal logistic regression model, this relationship was not significant. The results of a study of Iranian university students showed that the happiness scores of the students with hookah use experience and regular hookah smokers were lower than those of other students [24]. Nevertheless, a study in the US found no significant differences between hookah smokers and those with no hookah use experience in terms of the feeling of happiness [25]. One of the limitations of the present study is its cross-sectional nature and the impossibility of investigating the causality of relationships between the variables. Another limitation is that the study was limited to 10th-grade high school students. Furthermore, despite our emphasis on the confidentiality of their responses and the anonymity of questionnaires, the students’ self-reporting of their tobacco smoking status can be considered another limitation of the present study.

In conclusion, the results of the present study indicate that happiness scores were negatively associated with cigarette smoking stages among high school students. When controlling for other variables in the model, no association was found between happiness and hookah smoking. This underscores the necessity of conducting longitudinal or interventional studies in order to determine the effect of enhancing happiness on preventing the transition through the stages of cigarette and hookah smoking. Accordingly, it may be possible to provide purposeful programs for improving happiness in schools and within families in order to facilitate the prevention and cessation of cigarette and hookah smoking among students.

## Figures and Tables

**Table 1.** Demographic and other characteristics of the students according to cigarette smoking status

Characteristics	Never	Experimenter	Regular smoker	Total	p-value
Gender					
Men	404 (71.5)	101 (17.9)	60 (10.6)	565 (50.0)	<0.001
Women	486 (85.9)	73 (12.9)	7 (1.2)	566 (50.0)	
Total	890 (78.7)	174 (15.4)	67 (5.9)	1,131 (100.0)	
General risk-taking behaviors					
No	310 (93.1)	16 (4.8)	7 (2.1)	333 (28.7)	<0.001
Yes	522 (71.9)	150 (20.7)	54 (7.4)	726 (71.3)	
Experience of self-injury					
No	799 (82.7)	125 (12.9)	42 (4.3)	966 (90.7)	<0.001
Yes	40 (40.4)	39 (39.4)	20 (20.2)	99 (9.3)	
Substance abuse					
No	842 (79.1)	165 (15.5)	58 (5.4)	1,065 (99.2)	<0.001
Yes	2 (22.2)	2 (22.2)	5 (55.6)	9 (0.8)	
Having smoker friend(s)					
No	696 (89.1)	75 (9.6)	10 (1.3)	781 (74.5)	<0.001
Yes	137 (51.3)	82 (30.7)	48 (18.0)	267 (25.5)	
Age (yr)					
14-15	451 (78.2)	93 (16.1)	33 (5.7)	577 (51.3)	0.58
16-17	438 (80.1)	77 (14.1)	32 (5.9)	547 (48.7)	
Hookah smoking status					
Never	614 (92.9)	41 (6.2)	6 (0.9)	661 (61.7)	<0.001
Experimenter	218 (60.7)	108 (30.1)	33 (9.2)	359 (33.5)	
Regular hookah smoker	12 (23.1)	17 (32.7)	23 (44.2)	52 (4.8)	
Socioeconomic status					
Very low	217 (79.2)	47 (17.2)	10 (3.6)	274 (27.4)	0.08
Low	172 (78.2)	33 (15.0)	15 (6.8)	220 (22.0)	
Middle	154 (83.2)	26 (14.1))	5 (2.7)	185 (18.5)	
High	139 (78.1)	26 (14.6)	13 (7.3)	178 (17.8)	
Very high	103 (73.0)	22 (15.6)	16 (11.3)	141 (14.1)	
Smoker(s) in family					
No	572 (83.4)	86 (12.5)	28 (4.1)	686 (63.9)	<0.001
Yes	272 (70.3)	80 (20.7)	35 (9.0)	387 (36.1)	
Living with parents					
No	42 (5.0)	14 (8.4)	8 (12.5)	64 (6.0)	0.02
Yes	798 (95.0)	153 (91.6)	56 (87.5)	1,007 (94.0)	
Previous year average grades	18.48±1.31	18.22±1.20	17.51±1.70	18.38±1.34	<0.001
Attitude toward smoking	-10.51±2.48	-6.87±4.16	-3.22±4.45	-9.52±3.58	<0.001
Happiness score	124.14±19.47	112.82±20.43	113.49±18.53	121.74±20.08	<0.001

Values are presented as number (%) or mean±standard deviation.

**Table 2.** Demographic and other characteristics of the students according to hookah smoking status

Characteristics	Never	Experimenter	Regular hookah smoker	Total	p-value
Gender					
Men	284 (52.7)	203 (37.7)	52 (9.6)	539 (49.9)	<0.001
Women	380 (70.4)	158 (29.3)	2 (0.4)	540 (50.0)	
Total	664 (61.5)	361 (33.5)	54 (5.0)	1,079 (100.0)	
General risk-taking behaviors					
No	269 (79.8)	66 (19.6)	2 (0.6)	337 (31.7)	<0.001
Yes	386 (53.1)	294 (40.4)	47 (6.5)	727 (68.3)	
Experience of self-injury					
No	635 (65.4)	299 (30.8)	37 (3.8)	971 (90.7)	<0.001
Yes	25 (25.3)	59 (59.6)	15 (15.2)	99 (9.2)	
Substance abuse					
No	663 (62.0)	358 (33.5)	49 (4.6)	1,070 (99.2)	<0.001
Yes	1 (11.1)	3 (33.3)	5 (55.6)	9 (0.8)	
Having smoker friend(s)					
No	568 (72.4)	206 (26.3)	10 (1.3)	784 (74.5)	<0.001
Yes	87 (32.5)	145 (54.1)	36 (13.4)	268 (25.5)	
Age (yr)					
14-15	337 (61.2)	190 (34.5)	24 (4.4)	551 (51.4)	0.65
16-17	326 (62.6)	168 (32.2)	27 (5.2)	521 (48.6)	
Smoker(s) in family					
No	461 (67.1)	199 (29.0)	27 (3.9)	687 (63.7)	<0.001
Yes	203 (51.9)	161 (41.2)	27 (6.9)	391 (36.3)	
Socioeconomic status					
Very low	191 (69.7)	74 (27.0)	9 (3.3)	274 (27.4)	<0.001
Low	138 (62.2)	81 (36.5)	3 (1.4)	222 (22.2)	
Middle	111 (60.0)	69 (37.3)	5 (2.7)	185 (18.5)	
High	103 (57.5)	63 (35.2)	13 (7.3)	179 (17.9)	
Very high	71 (50.7)	54 (38.6)	15 (10.7)	140 (14.0)	
Cigarette smoking status					
Never	614 (72.7)	218 (25.8)	12 (1.4)	844 (78.7)	<0.001
Experimenter	41 (24.7)	108 (65.1)	17 (10.2)	166 (15.5)	
Regular smoker	6 (9.7)	33 (53.2)	23 (37.1)	62 (5.8)	
Living with parents					
No	33 (50.8)	26 (40.0)	6 (9.2)	65 (6.0)	0.10
Yes	629 (62.2)	334 (33.0)	48 (4.7)	1,011 (94.0)	
Previous year average grades	18.54±1.24	18.24±1.46	17.6±1.75	18.40±1.36	<0.001
Attitude toward smoking	-10.52±2.63	-8.27±4.08	-5.3±4.74	-9.50±3.60	<0.001
Happiness score	123.82±20.21	118.21±19.40	118.51±19.53	121.7±20.08	<0.001

Values are presented as number (%) or mean±standard deviation.

**Table 3.** Ordinal logistic regression analysis of the relationships of cigarette smoking and hookah smoking status with risk variables

Variables	Cigarette smoking		Hookah smoking	
	OR (95% CI)	p-value	OR (95% CI)	p-value
Gender, men	2.62 (1.34, 5.13)	0.005	1.57 (1.07, 2.30)	0.52
Exhibiting general risk-taking behavior	1.69 (0.95, 2.98)	0.07	2.16 (1.50, 3.10)	<0.001
Having self-injury	1.46 (0.84, 2.54)	0.18	1.80 (1.09, 2.97)	0.02
Substance abuse	3.22 (0.57, 18.15)	0.18	3.10 (0.65, 14.87)	0.16
Having smoker friend(s)	2.38 (1.51, 3.75)	<0.001	2.01 (1.38, 2.92)	<0.001
Hookah smoking status				
Never	1.00 (reference)		-	-
Experimenter	4.75 (3.00, 7.51)	<0.001	-	-
Regular hookah smoker	14.81 (6.18, 35.53)	<0.001	-	-
Cigarette smoking status				
Never	-	-	1.00 (reference)	
Experimenter	-	-	4.45 (2.88, 6.89)	<0.001
Regular smoker	-	-	8.25 (3.87, 17.63)	<0.001
Socioeconomic status				
Very low	1.00 (reference)		1.00 (reference)	
Low	0.80 (0.45, 1.43)	0.45	1.26 (0.81, 1.97)	0.31
Middle	0.41 (0.22, 0.78)	0.007	1.75 (1.11, 2.78)	0.02
High	0.64 (0.33, 1.24)	0.19	1.69 (1.05, 2.71)	0.03
Very high	0.34 (0.16, 0.69)	0.003	2.00 (1.20, 3.36)	0.008
Smoker(s) in family	1.73 (1.14, 2.61)	0.01	1.24 (0.91, 1.69)	0.18
Living with parents	0.44 (0.21, 0.91)	0.03	0.98 (0.53, 1.83)	0.95
Previous year average grades (higher grades)	1.00 (0.84, 1.20)	0.98	0.96 (0.85, 1.10)	0.57
Attitude toward smoking (positive scores)	1.34 (1.26, 1.42)	<0.001	1.05 (0.99, 1.10)	0.58
Happiness score (higher scores)	0.99 (0.97, 0.99)	0.01	10.1 (0.97, 1.02)	0.52

OR, odds ratio; CI, confidence interval.
